# cPlot: Contig-Plotting Visualization for the Analysis of Short-Read Nucleotide Sequence Alignments

**DOI:** 10.3390/ijms231911484

**Published:** 2022-09-29

**Authors:** Mingeun Ji, Yejin Kan, Dongyeon Kim, Jaehee Jung, Gangman Yi

**Affiliations:** 1 Department of Multimedia Engineering, Dongguk University, Seoul 04620, Korea; 2 Department of Information and Communication Engineering, Myongji University, Yongin-si 17058, Korea

**Keywords:** contig plotting, read alignment, sequence alignment

## Abstract

Advances in the next-generation sequencing technology have led to a dramatic decrease in read-generation cost and an increase in read output. Reconstruction of short DNA sequence reads generated by next-generation sequencing requires a read alignment method that reconstructs a reference genome. In addition, it is essential to analyze the results of read alignments for a biologically meaningful inference. However, read alignment from vast amounts of genomic data from various organisms is challenging in that it involves repeated automatic and manual analysis steps. We, here, devised cPlot software for read alignment of nucleotide sequences, with automated read alignment and position analysis, which allows visual assessment of the analysis results by the user. cPlot compares sequence similarity of reads by performing multiple read alignments, with FASTA format files as the input. This application provides a web-based interface for the user for facile implementation, without the need for a dedicated computing environment. cPlot identifies the location and order of the sequencing reads by comparing the sequence to a genetically close reference sequence in a way that is effective for visualizing the assembly of short reads generated by NGS and rapid gene map construction.

## 1. Introduction

With the advances in next-generation sequencing (NGS) technology, biologists have laid a quantitative framework for conducting genome research by accumulating vast amounts of genomic data from various organisms in text form. However, the assembly of sequences generated by NGS requires a large computation effort focused on comparing very short DNA sequences. Further, performing read alignment using traditional dynamic programming-based read alignment algorithms is difficult. Thus, various read alignment methods based on seed extension, hash, and suffix trees have been proposed.

Examples of the currently used alignment programs include MAQ [1], Stampy [2], and ntHash [3]. While these tools generate hash tables and functions for short-read sorting, their applicability for read alignment has also been investigated. For instance, MAQ performs read alignment by constructing short reads of the query sequence as a hash table and searching for reference sequences [1]. However, rapid sorting is difficult to achieve, because the hash table for each query sequence has to be newly constructed by read-hashing based on the query sequence rather than a reference sequence, and the reference sequence has to be scanned multiple times. As another example, Stampy performs read alignment using a read search method, with a hash table constructed by hashing a *k*-mer-sized sequence from a reference sequence [2]. Therein, short reads are aligned by generating all sequences that can be assembled within one mismatch error. However, with repeated reads, the mapping speed is greatly reduced because of the chaining problem in the hash table. Finally, ntHash [3] and MHAP [4] were proposed as hashing methods to improve the performance of the hashing algorithm by recursively calculating the hash value for the nearby *k*-mer in the query sequence. Of note, the existing short-read alignment methods involve a tedious manual comparison [5] of results obtained by various calculation methods, according to data properties, for an optimized read alignment output [6].

Most short-read alignment studies [7,8,9] of vast amounts of genomic data involve comparisons of text-based data. However, visual inspection of large amounts of generated data is time-consuming [10]. To address this, tools for rapid read alignment and visual result analysis have been developed. For example, mummerplot [11,12] is a tool for a rapid alignment of DNA sequences, which can perform seed extension-based alignment, such as BLAST [13], quickly align the data into 20 bp units using the suffix tree algorithm, and produce visual outputs using data visualization tools, such as gnuplot [14]. r2cat [15] matches a set of contigs to the reference genome, aligns the matched contigs, and plots the results. Another example, D-Genies [16], was proposed for pairwise-based interactive plotting; however, it cannot be used to compare the alignment results for multiple query sequences. Accordingly, we, here, propose cPlot, an application for aligning reads generated by sequence assembly, by comparison with genetically close references. cPlot, which is an improvement on the existing read alignment methods dedicated to similarity analysis of mixed small-fragment reads, allows the user to intuitively check the results by aligning the similarity of different sequences and providing a visualization analysis interface. The final visualized results are provided in a web-based interface environment. In addition, several options are provided for sorting and comparing various sequences, and the user can check all previously performed cPlot tasks in a web browser, using the user history. The software greatly facilitates short-read alignment and will improve to identify the location and order of the sequencing reads by comparing the sequence to a reference sequence. This is an effective approach for the assembly of short reads generated by NGS and rapid gene map construction. The software can be accessed at https://datalab.dongguk.edu/cPlot, accessed on 18 August 2022.

## 2. Results and Discussion

The performance of cPlot was verified to ensure accurate read alignment of a single reference sequence and multiple query sequences, and to check optimal alignment of small, scattered read fragments. To achieve this, first, the query sequences (generated by splitting the reference sequence into multiple small fragments that were then randomly shuffled) and the original reference sequence were aligned. This enabled verification of whether an accurate alignment of sequences that are almost identical is possible in cPlot. Next, the reads of the aligned small fragments were optimally aligned to determine whether they could be reassembled into the same sequence as the reference sequence. Finally, the results were compared with those obtained using another algorithm. Details of the analysis are presented below.

### 2.1. Dataset

Table 1 shows the dataset used. It contains one reference sequence and eight query sequences. One query sequence is the same sequence as the reference sequence. The eight sequences are plastid genomic sequences from Cryptophyta, with repeats between positions 20,000 bp and 40,000 bp [17]. A well-known conventional algorithm identified repeats in only some regions of these sequences. This dataset was therefore ideal for testing the performance of cPlot.

### 2.2. Identifying Optimal k-mer Size for Improved Performance

An appropriate *k*-mer size is required for improved alignment results from contig plotting. With a dataset where the reference sequence is almost identical to the query sequence, similar alignments can be obtained regardless of the *k*-mer value, and the higher the *k*-mer value, the shorter the alignment time. By contrast, in a dataset in which the reference sequence and the query sequence are dissimilar, an optimal *k*-mer size should be determined to achieve a relatively short alignment time and high-similarity alignment. In general, it is difficult to determine the degree of similarity between a reference sequence and a query sequence. For the analyzed dataset (Table 1), the optimal *k*-mer size was determined by comparing the sequence similarity and alignment time for *k*-mer sizes 10–50 bp. The accuracy of read alignment was based on the ratio of the length of sequence obtained as the optimal result to that of the existing reference sequence. For an accurate similarity analysis, read alignment should be performed using a small *k*-mer value. However, the computation load rapidly increases below a specific *k*-mer value, which can be problematic. On the other hand, with a large *k*-mer value, the reads are rapidly aligned in a high-throughput manner, but the detected similarity decreases as the number of *k*-mer-sized reads that allow for a possible alignment is dramatically reduced.

### 2.3. Accuracy of Contig Plotting

**Figure 1:**
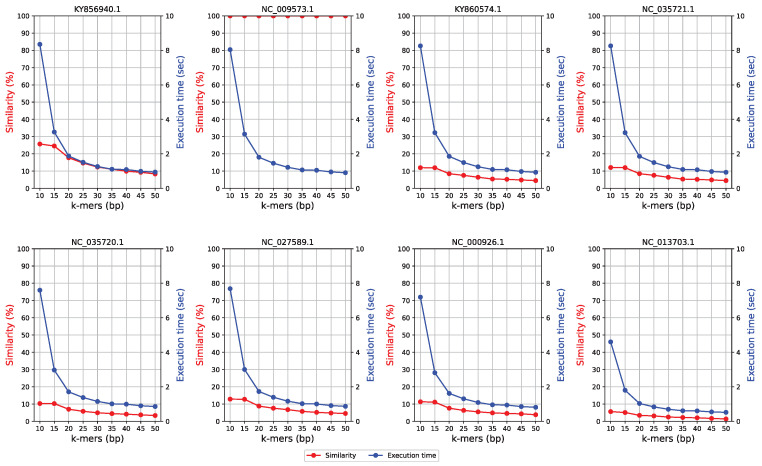
Similarity and execution time of read alignment by *k*-mer size.

Figure 1 shows the results of similarity determination and execution time for the read alignment of each sequence from the dataset presented in Table 1. The similarity of read alignments of the eight query sequences was comparable for the *k*-mer values of 10 to 15; however, as the *k*-mer value decreased, the execution time inversely increased. Therefore, in the current study, the appropriate *k*-mer value was set to 15. For query sequence NC 009573.1, which was identical to the reference sequence, the similarity value was high regardless of the *k*-mer size. Therefore, when aligning two similar sequences, the alignment can be performed in a high-throughput manner by increasing the *k*-mer value. The query sequence was a reference sequence that had been split into 1, 2, 4, 6, 8, and 10 fragments, and randomly reassembled. The accuracy of read alignment of the reference sequence and the randomly rearranged query sequence was more than 99.99%. In the current study, the read alignment of the uncertain base “N” was not considered. Read alignment based on the reference sequence from which the uncertain base “N” was removed resulted in 100% accurate alignment. However, during reassembly after fragmentation, the *k*-mer-sized reads generated at sequence positions before splitting were not considered for read alignment.

**Figure 2:**
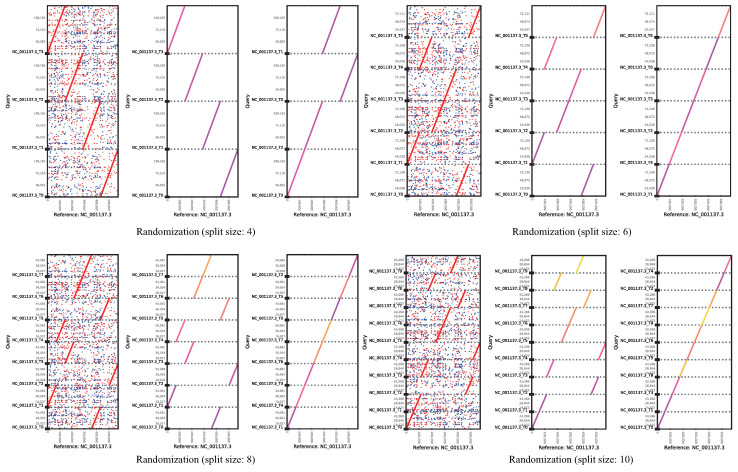
Comparative visualized analysis of short-read alignments with a split size.

To determine the accuracy of the cPlot algorithm, the query sequence was divided and shuffled into fragments of arbitrary sizes, and matched with the original reference sequences. Figure 2 shows the results of short-read alignment of a reference sequence (NC_001137.3) and query sequences after shuffling; each x-axis is the base position of the reference sequence and the y-axis is the base position of the reassembled query sequence. The *k*-mer size for each split size was fixed at 15. The read alignment was accurate even after dividing into several files and reassembling in random order. Table 2 shows the sorted length and similarity for each query sequence with a split size of 8 in Figure 2. That is, when read alignment of the reassembled reference sequence was performed, the dreads were correctly aligned with 99.99% similarity. The aligned length of each query sequence was 2–3 bp shorter than the total length, because the reads generated in the section dividing the reference sequence were removed. A similarity of 99.99% or more was obtained even after excluding the read alignment for the segment to be divided. The “N” in the undefined reference sequence was not considered during read alignment.

### 2.4. Comparative Results

**Figure 3:**
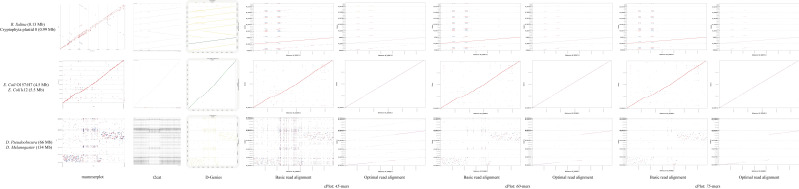
Comparative visualized results for mummerplot, r2cat, D-Genies, and cPlot.

The results of mummerplot, r2cat, D-Genies, and the basic read alignment of proposed cPlot were compared (Figure 3). We selected genomes of various sizes from NCBI and used them as a dataset because the size of the genome affects the performance of alignment. Since cPlot is based on *k*-mer, we show the results for various *k*-mer values for more accurate performance comparison. Through this comparative analysis, it can be confirmed that cPlot provides high-accuracy alignment results compared to other algorithms.

The mummerplot in Figure 3 has the problem of overlapping the alignment results of the Cryptophyta plastid genome, which is less than 1 Mb, while cPlot shows identifiable alignment results for the same dataset, and has no deviation in various *k*-mer values. cPlot and r2cat align relative regions accurately in a genome of less than 10 Mb. However, r2cat has the limitation that represents unidentifiable results in the large D. Pseudoobscura genome. However, cPlot clearly identifies the alignment region in the same case, and has high performance, especially if the *k*-mer value is large in proportion to the genome.

**Figure 4:**
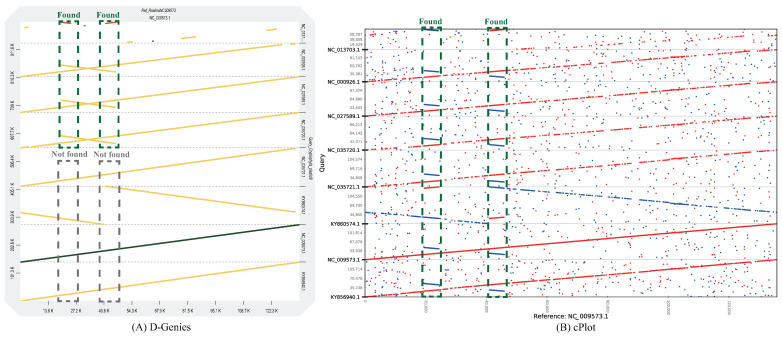
Comparative visualized analysis for repeat region. (**A**) D-Genies represents from 20,000 bp to 40,000 bp of four Cryptophyta plastid genomes as one alignment. (**B**) cPlot identifies repeat regions at 20,000 bp and 40,000 positions in all Cryptophyta plastid genomes.

Short-read alignment has limitations in identifying repeat regions due to read length constraints. This means that identifying more candidates of repeat regions provides better performance. We performed the comparative analysis of cPlot (Figure 4). Although D-Genies represents more accurate results (Figure 3) overall than mummerplot and r2cat in genomes of various sizes, it only identifies some repeat regions (Figure 4A). On the other hand, repeat regions show that cPlot (Figure 4B) finds and indicates more repeats at positions 20,000 bp and 40,000 bp. These results show that cPlot outperforms other algorithms, and it derives meaningful results for further analysis on the same dataset.

We proved the alignment performance of cPlot through Figure 3 and Figure 4. After the basic read alignment, cPlot performed optimal read alignment and rearranging for multiple query sequences. These additional steps selected the optimal read set based on the overlap information and, finally, visualized it by aligning the similarity score. It can be seen that numerous noises observed in mummerplot, r2cat, and D-Genies are removed in cPlot (Figure 3). Since cPlot provides visualized results after sorting, it enables users to intuitively confirm results and provides meaningful results for similarity analysis between sequences.

## 3. Materials and Methods

### 3.1. Algorithm

**Figure 5:**
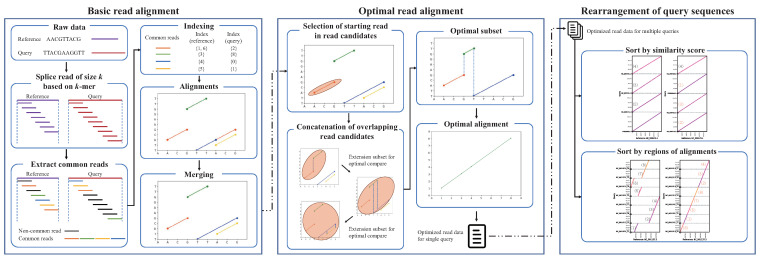
Overview of the cPlot approach.

Several methods for read alignment have been proposed to date, but some analytical issues persist, such as the failure to sort regions of repeat sequences. Further, a method that would enable the user to visually analyze large amounts of data is needed, since the results are derived from a comparative analysis of text-based data. Accordingly, we propose cPlot, which performs read alignments by considering all the possible combinations of sequences, as well as allowing the user to intuitively check the contig-plotting results. It performs sequence alignment and provides visualized contig-plotting output by relying on an algorithm that incorporates three components: basic read alignment, optimal read alignment, and query sequence rearrangement, as shown in Figure 5.

#### 3.1.1. Basic Read Alignment

The first step of analysis is “basic read alignment”. Here, a DNA sequence file is used as an input file to align single- or multiple-query sequences with a single reference sequence. The aligned reads are then *k*-mer-sized and indexed. The number of indexed reads generated by the *k*-mer value determines the alignment speed. A small value means indexing increases and sorting is sensitive, whereas a large value means the indexing number is relatively small, leading to less sensitive sorting. If the two sequences to be compared are similar, the sorting is rapid.

#### 3.1.2. Optimal Read Alignment

The second step of analysis is “optimal read alignment”. In this step, in order to intuitively analyze the similarity between the reference sequence and the query sequence, reads with a long length and close to the region of the reference sequence are optimally selected and aligned based on the region of the reference sequence. During the first basic alignment step, only reads with overlaps between the reference sequence and query sequence are aligned [18]. Read alignment results in the reads overlapping in a specific region of the reference sequence, and an additional analysis step is needed to accurately select the reads most similar to the reference sequence. Therefore, to select the candidate read that is most similar to the reference, in the second analysis step, the single sequence that is most similar to the reference sequence region and with the longest base alignment is identified among the reads sorted based on the index during the first analysis step. Based on the resulting read sets generated during the first step, the algorithm loops from the read closest to the region at the start of the reference sequence and collates the overlapping reads into a subset. From the subset of reads, a candidate set (consisting of the reads that are most similar to the reference sequence region, while having the highest alignment score) is selected for optimal alignment. The smaller the difference between each overlapping sequence, the greater its similarity to the reference sequence region. After analyzing all the overlapping regions, the reads included in the candidate set are sorted based on the reference sequence region.

#### 3.1.3. Query Sequence Rearrangement

In the last step of analysis, when multiple query sequences are input, the order of the queries is rearranged based on similarity to the reference sequence. In this step, the analysis of one query sequence is the same as the result of “optimal read alignment”. Read alignment incorporates two functions. First, multiple query sequences are automatically sorted based on similarity to the reference sequence, from the most similar to the least similar sequence. Then, several query sequences mapped to the reference sequence region are automatically sorted into regions similar to the reference. Specifically, this function automatically sorts various scattered query sequences and rearranges them in a sequence similar to the reference sequence. Regardless of the input order, the query sequences are rearranged considering the appropriate region of the mapped sequence, with no need for a manual rearrangement and analysis of the query sequence when conducting an existing read alignment.

### 3.2. Application

#### 3.2.1. Web-Based Interface for Contig Plotting

**Figure 6:**
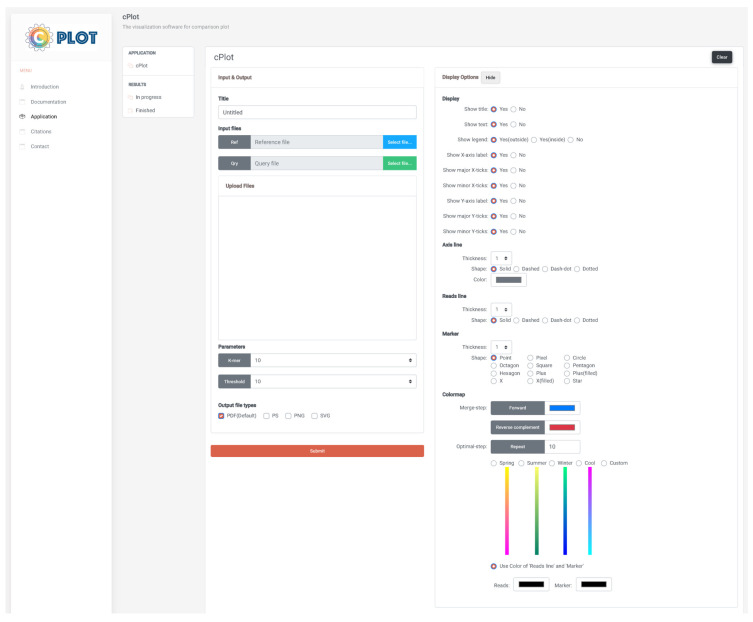
cPlot web page.

The core engine of cPlot is based on Python, and the web-based user interface was implemented with Bootstrap, JavaScript, PHP, and matplotlib [19]. The web-based interface enables easy input and submission of tasks, as well as result viewing. As an example, Figure 6 shows the web-based interface that allows the user to perform contig plotting. The user can easily submit and manage contig-plotting tasks in cPlot, without having to build a separate environment.

#### 3.2.2. Input Data Required for Contig Plotting

The following input configurations are accounted for in cPlot: the input file, parameter, output file type, and display. cPlot uses FASTA format files (with .fasta, .fa, .fsa, and .fna extensions as inputs) that include multiple reads to compare sequence similarity. The user must upload one reference sequence file and at least one multiple-query sequence file as the “input file” in a system configured within a web environment. “Options” directly affects contig plotting. The options include *k*-mer size and threshold. The former defines the size of fragments into which each read is split, followed by their sorting. A small *k*-mer value may increase the accuracy of the read alignment, but the corresponding execution time may increase because of increased calculations. To address this, an appropriate *k*-mer value must be specified. In the current study, the optimal *k*-mer value was 15. The threshold option is the minimum length (in bp) to be printed in contig plotting. The value must be greater than or equal to the *k*-mer value; a greater value removes the generated default reads.

**Figure 7:**
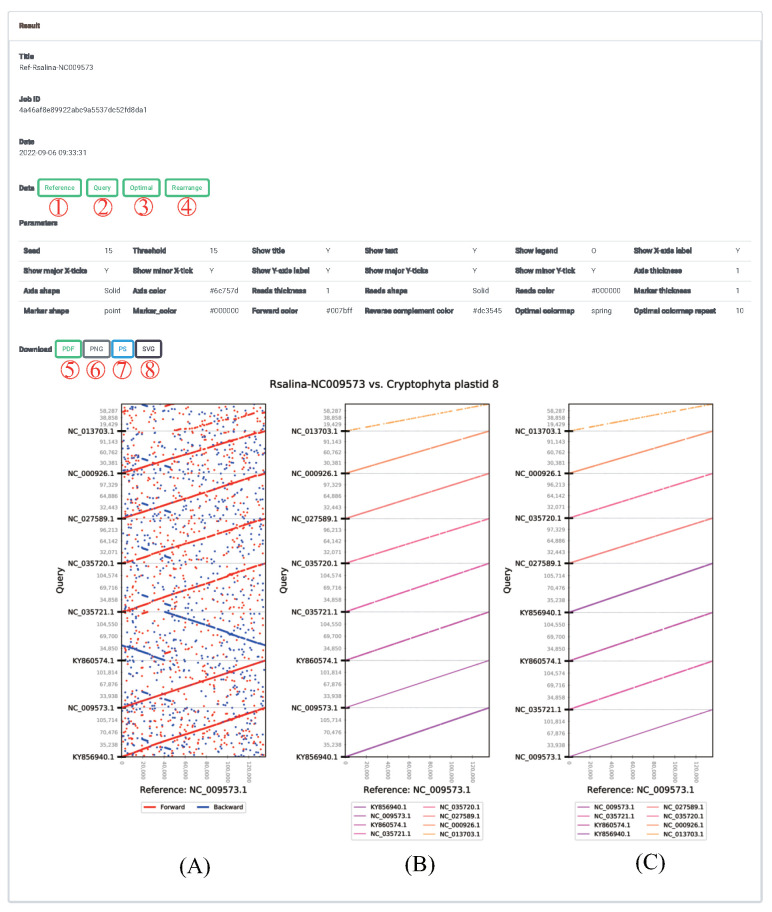
Submitted job results. ①: Reference sequence file uploaded by the user. In (**A**–**C**), the x-axis refers to the reference sequence. ②: Query sequence uploaded by the user. The input files are sequentially merged and provided as a single file. In (**A**–**C**), the y-axis refers to the query sequence. ③: Based on the position of the reference sequence, modified positions and sequence information for the optimized query sequence can be checked based on the text. ④: Query sequences with a low similarity to the reference sequence can be viewed in a top-down format, in ascending order. ⑤–⑧: The generated cPlot results that can be downloaded in .pdf, .png, .ps, and .svg formats (download extension option is selected prior to cPlot execution).

Figure 7 shows the cPlot output, which can be checked using a web-based interface, and provides an overview of the data used in a job and results files. The file contents and results of each data analysis type are described below. Figure 7A–C show the results of automatic analysis in cPlot, presenting significant similarity between sequences. Figure 7A shows typical read alignment results. By comparing with the reference sequence, for each sequence in a multiple-query sequence, short reads of a fixed *k*-mer size that can be combined into longer reads are merged, and the final aligned reads of *k*-mer size are displayed on the screen. To obtain all sortable reads in the two sequence directions (5′ → 3′ and 3′ → 5′), cPlot performs read alignment by generating a general query sequence in the 5′ → 3′ direction and a reverse complemented query sequence in the 3′ → 5′ direction, based on the single/multiple query sequence input. In Figure 7A, the reads in the 5′ → 3′ direction are indicated in blue, and those in the 3′ → 5′ direction are shown in red.

**Figure 8:**
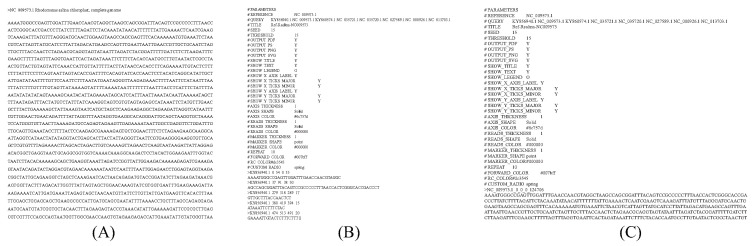
Sample input file and the resultant output file. (**A**) Input file (nucleotide.fasta). (**B**) Output file that aligned sequence from the optimal alignment step. (**C**) Output file that rearranged query sequences.

Figure 7B shows the results of optimal alignment of reads from Figure 7A. Comparing the resulting reads in Figure 7A with the reference sequence, only the most significant reads are extracted, and reads overlapping with the extracted reads are removed. For each query sequence, significant reads are those that match the longest base sequence at a reference sequence position. In addition, the significant read sequence is a query sequence corresponding to the reference sequence, and may be highly similar to the query sequence. Therefore, the query sequence is assembled based on the reference sequence position to generate an optimal query sequence. The optimal query sequence is provided in Figure 8B.

Figure 7C shows the rearranged optimal query sequence based on weight. In the optimal alignment result, weight is given to a portion of the reference sequence and the aligned sequence, and the query sequences with high similarity to the reference sequence are sorted in the bottom-up format, in descending order. In Figure 7A,B, the reference sequence is divided and ordered based on the order of the random input sequence, so that the query sequence similar to the reference sequence is arbitrarily scattered. Output file type provides an option to select the extension of the image file for the contig-plotting results. The output types provide .pdf, .ps, .png, and .svg extensions to store the contig-plotting results. By default, an image file with a .pdf extension is generated. The display option can be used to specify the properties of the resulting plot in the plotting option, such as the title, color for each query sequence, x-axis label, y-axis label, legend, point size, and line thickness. The user can generate various images by modifying the default options.

Figure 8 shows the text forms of the input and output files. Figure 8A is an example of a FASTA file generated by the user in cPlot. Only the FASTA file can be used as input data for read alignment. Figure 8B, an example of Figure 7-③ conversion, shows the sequence name, initial position, optimally aligned position, and sequence. Figure 8C, an example of Figure 7-④ conversion, shows the sequences sorted from the most similar matched sequence down, based on the overall query sequence. The aligned sequences in Figure 8C were generated based on the optimally ordered query sequence in Figure 7C.

#### 3.2.3. Job Management

After specifying the input sequence file and user-defined options (Figure 6), cPlot is launched using the [SUBMIT] button. The job list is a list of performed cPlot jobs. Basic information about each job can be obtained therein, with the result for each extension easily downloaded. Detailed information about each job is checked using the [RESULT] button in the URL column.

## 4. Conclusions

Identification of the location and order of the sequencing reads by comparing the sequence to a reference sequence is an effective approach for the assembly of short reads generated by NGS and rapid gene map construction. Many alignment algorithms have been developed, but most of them focus on sequence region matching or alignment. cPlot, the software developed in the current study, is a visual tool for read sequence alignment that allows efficient evaluation of repeat analyses by performing index-based read alignment. Further, cPlot provides a web-based environment for basic read alignment that reflects the optimal region and query sequence rearrangements for all reads of a specified *k*-mer size, including specific nucleotide sequences, such as repetitive sequences. We expect that cPlot will enable biologists to facilitate studies that require short sequence readouts and alignment to novel genomes.

## Figures and Tables

**Table 1:** Dataset.

Category	Sequences
Reference sequence	*Rhodomonas salina* (NC_009573.1)
Query sequence	Cryptophyta plastid 8 ^1^

^1^ KY856940.1, NC_009573.1, KY860574.1, NC_035721.1, NC_035720.1, NC_027589.1, NC_000926.1, and NC_013703.1.

**Table 2:** Similarity of read alignments per divided sequence.

Name	Length (bp)	Mapped Contigs (bp)	Similarity (%)
NC_001137.3_T0	72,100	72,098	99.997
NC_001137.3_T1	72,100	72,098	99.997
NC_001137.3_T2	72,100	72,098	99.997
NC_001137.3_T3	72,100	72,098	99.997
NC_001137.3_T4	72,100	72,097	99.995
NC_001137.3_T5	72,100	72,097	99.995
NC_001137.3_T6	72,100	72,098	99.997
NC_001137.3_T7	72,174	72,171	99.995
Total	576,874	576,855	99.996

## Data Availability

Not applicable.
